# PD-1 blockade and CDK4/6 inhibition augment nonoverlapping features of T cell activation in cancer

**DOI:** 10.1084/jem.20220729

**Published:** 2023-01-23

**Authors:** Lestat R. Ali, Ana C. Garrido-Castro, Patrick J. Lenehan, Naima Bollenrucher, Courtney T. Stump, Michael Dougan, Shom Goel, Geoffrey I. Shapiro, Sara M. Tolaney, Stephanie K. Dougan

**Affiliations:** 1 Department of Cancer Immunology and Virology, Dana-Farber Cancer Institute, Boston, MA, USA; 2 Department of Medical Oncology, Dana-Farber Cancer Institute, Boston, MA, USA; 3 Department of Immunology, Harvard Medical School, Boston, MA, USA; 4 Department of Medicine, Harvard Medical School, Boston, MA, USA; 5 Department of Medicine, Division of Gastroenterology, Massachusetts General Hospital, Boston, MA, USA; 6 Peter MacCallum Cancer Centre, Melbourne, Australia; 7 The Sir Peter MacCallum Department of Oncology, University of Melbourne, Melbourne, Australia

## Abstract

We performed single-cell RNA-sequencing and T cell receptor clonotype tracking of breast and ovarian cancer patients treated with the CDK4/6 inhibitor ribociclib and PD-1 blockade. We highlight evidence of two orthogonal treatment-associated phenomena: expansion of T cell effector populations and promotion of T cell memory formation. Augmentation of the antitumor memory pool by ribociclib boosts the efficacy of subsequent PD-1 blockade in mouse models of melanoma and breast cancer, pointing toward sequential therapy as a potentially safe and synergistic strategy in patients.

## Teaser

The authors use single-cell RNA-sequencing and TCR tracking to analyze blood and tumors from breast and ovarian cancer patients treated with PD-1 blockade and CDK4/6 inhibition. They find that both therapies are immunologically active and enhance T cell effector function and memory, respectively.

## Introduction

Inhibitors of Cyclin-Dependent Kinases 4 and 6 (CDK4/6) have recently become keystone agents in the treatment of advanced hormone-receptor-positive breast cancer (Fassl et al., 2022). Beyond their effects on cell-cycle progression, they have been shown to function as potent modulators of T cell immunity (Deng et al., 2018; Goel et al., 2017; Schaer et al., 2018; Zhang et al., 2018). This insight has led to efforts exploring whether these agents can synergize with established T cell–targeting immunotherapies, such as blockers of the immune checkpoint Programmed Cell Death Protein 1 (PD-1; Tolba et al., 2021; Yuan et al., 2021).

Immune checkpoint blockade (ICB) fundamentally centers around the relief of effector T cell inhibition with antibodies targeting multiple inhibitory pathways now approved for clinical use (Burnell et al., 2022; Schildberg et al., 2016). Prior to the advent of ICB, diseases such as metastatic melanoma were uniformly lethal, whereas today ICB can induce a positive response in nearly half of treated patients (Larkin et al., 2019). However, the limited lifespan of an invigorated effector T cell cannot account for “tail of the curve” survivors, some of whom remain free of disease well beyond 10 yr (Wherry et al., 2003; Wolchok et al., 2022). This remarkable phenomenon depends on the persistence of long-lived antitumor memory T cells. CD8 T cell fate decisions are affected in the early stages of priming, with lineage commitment occurring prior to the first cell division (Clancy-Thompson et al., 2018; Kakaradov et al., 2017). Several factors have been shown to skew toward the memory cell fate including low antigen density, increased reliance on oxidative phosphorylation, and low Myc activity (Guo et al., 2022; Henrickson et al., 2013; Kaech and Ahmed, 2001; Pearce et al., 2009; van der Windt et al., 2012; Verbist et al., 2016). Despite the critical importance of memory cells, no therapies have been definitively shown to target or induce their formation. CDK4/6 inhibitors have emerged as promising candidates, with our group and others demonstrating that they enhance memory formation in mouse models of melanoma and in breast cancer patients (Heckler et al., 2021; Lelliott et al., 2021). We previously showed that exposure to CDK4/6 inhibitors at the time of T cell priming decreased activity of Myc and increased persistence of adoptively transferred tumor-specific T cells in mice. In humans, recently activated CD8 T cells from peripheral blood of patients starting on CDK4/6 inhibitors showed clonotype skewing toward memory precursors and decreased MYC activity, consistent with mechanistic studies performed in mice (Heckler et al., 2021).

High-grade toxicities often encountered upon combination treatment with a CDK4/6 inhibitor and PD-1 blockade have made it difficult to assess long-term clinical outcomes (Dougan et al., 2021; Pujol et al., 2021). Consequently, whether CDK4/6 inhibition acts cooperatively with PD-1 blockade in antitumor immunity has remained unclear. Here, we demonstrate by single-cell RNA-sequencing (scRNA-seq) of patient blood and tumor samples that dual treatment does affect distinct aspects of T cell activation, with PD-1 blockade invigorating short-term effector responses while CDK4/6 inhibition promotes the expression of markers of stemness and memory. Based on our findings, we proposed that sequential therapy may be a more optimal dosing strategy, whereby early treatment with a CDK4/6 inhibitor promotes the formation of a more durable memory T cell pool that can later serve as a substrate for PD-1 blockade, and we validate this hypothesis in a mouse model of melanoma of neoadjuvant treatment of melanoma and breast cancer.

## Results and discussion

**Figure 1.**
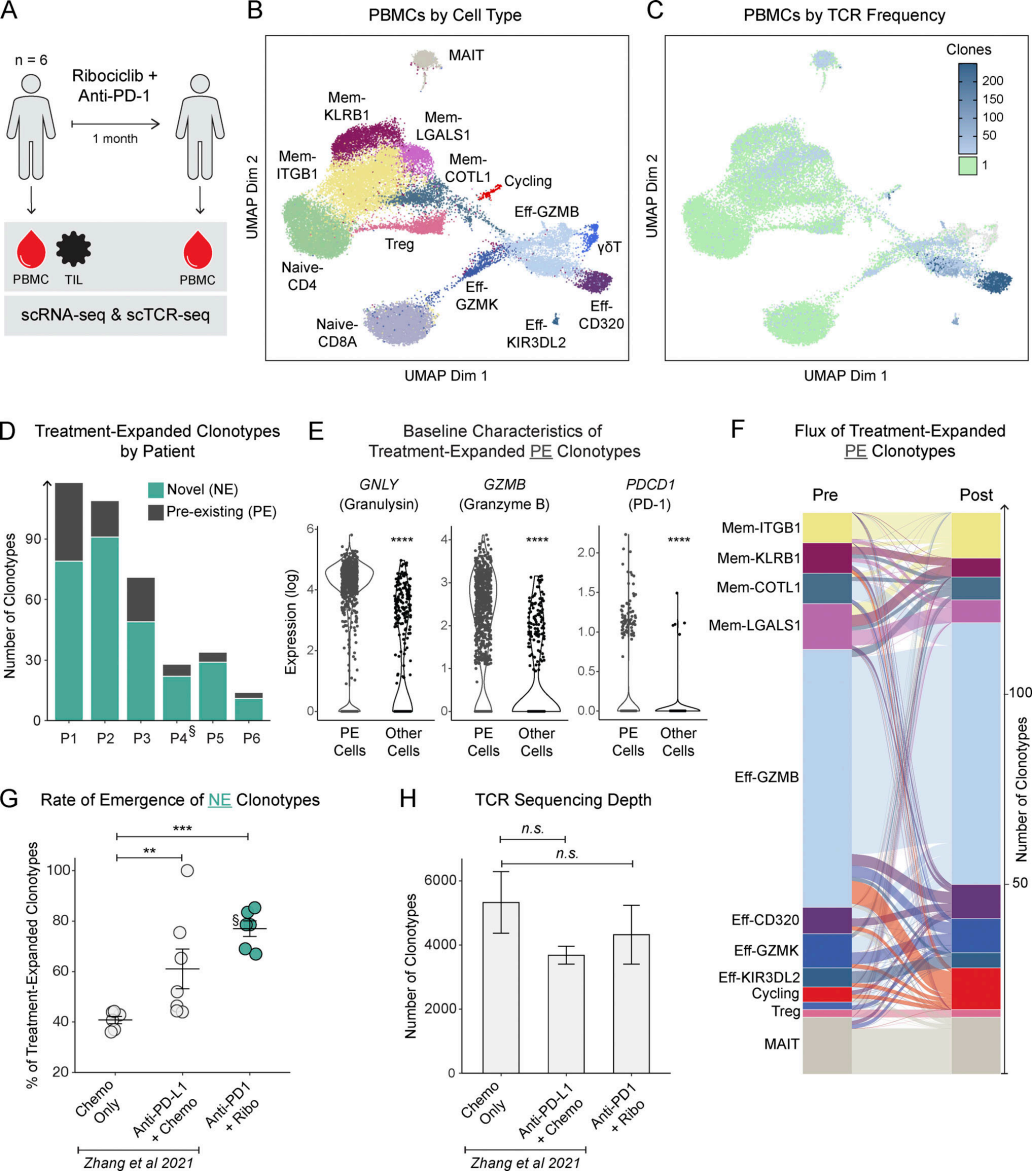
**Tracking circulating T cell clonotypes over time reveals clonal growth of pre-existing effector populations and the emergence of novel expanded clones.** PBMCs from metastatic breast or ovarian cancer patients investigationally treated with ribociclib and anti–PD-1 were sorted for CD3^+^ cells and analyzed by scRNA-seq and scTCR-seq. Cells expressing the same TCR sequence were grouped into clonotypes. **(A)** Schema of clinical trial and sample collection. **(B)** UMAP of cells colored by unsupervised clustering. **(C)** UMAP of cells colored by the observed frequency of their TCR. **(D)** Bar plot of the number of clonotypes in each patient that were clonally expanded (2+ cells) in post-treatment samples and observed at a higher frequency than in pre-treatment samples; clonotypes were distinguished based on whether they had been found in pre-treatment samples or not. The patient with ovarian cancer is marked by a section sign (§). **(E)** Log-normalized expression of key genes in pre-treatment cells belonging to clonotypes that expanded after treatment in comparison to pre-treatment cells belonging to clonotypes that were expanded at baseline but did not expand further after treatment. Each dot represents a cell. Wilcoxon rank-sum test. **(F)** Alluvial plot showing clonotype flux from pre-treatment to post-treatment clusters; the thickness of a band connecting two clusters represents the number of clonotypes whose member cells were found in the corresponding clusters. **(G)** Comparison of the frequency of novel expanded clonotypes by treatment. Each dot represents a different patient. Wilcoxon rank-sum test. **(H)** Bar plot of clonotype detection depth under the different treatment conditions in G. *N* = 6–7 per group. Wilcoxon rank-sum test. All error bars show mean ± SEM. **P <0.01, ***P <0.001, ****P <0.0001.

We sequenced circulating T cells and tumor-infiltrating lymphocytes (TILs) from six patients with metastatic breast or ovarian cancer enrolled in a phase I clinical trial (NCT03294694) investigating the combination of the CDK4/6 inhibitor ribociclib with spartalizumab, a monoclonal antibody against PD-1 (Fig. 1 A and Table 1). Samples were obtained before treatment and after one cycle of treatment. Single cells were also subject to TCR-seq, allowing us to group T cells expressing the same TCR into clonotypes and to track their fates across time and tissue site (Luoma et al., 2020).

**Table 1.** Clinical characteristics of patient cohort

Patient characteristics	N = 6
**Age, mean (SD)**	50.7 (12.3)
**Female sex**	6 (100%)
**Diagnosis**	
Breast cancer, HR+ HER2−	5 (83%)
Ovarian cancer	1 (17%)
**Stage**	
I–III	0 (0%)
IV	6 (100%)
**Treatment**	
Ribociclib + Anti–PD-1	1 (17%)
Ribociclib + Anti–PD-1 + Fulvestrant	5 (83%)
**Best overall response**	
Complete or partial response	0 (0%)
Stable disease <6 mo	3 (50%)
Progressive disease	3 (50%)
**Hepatotoxicity**	
Grade 3	3 (50%)
Grade 2	2 (33%)
Grade 1	0 (0%)
None	1 (17%)

**Figure S1.**
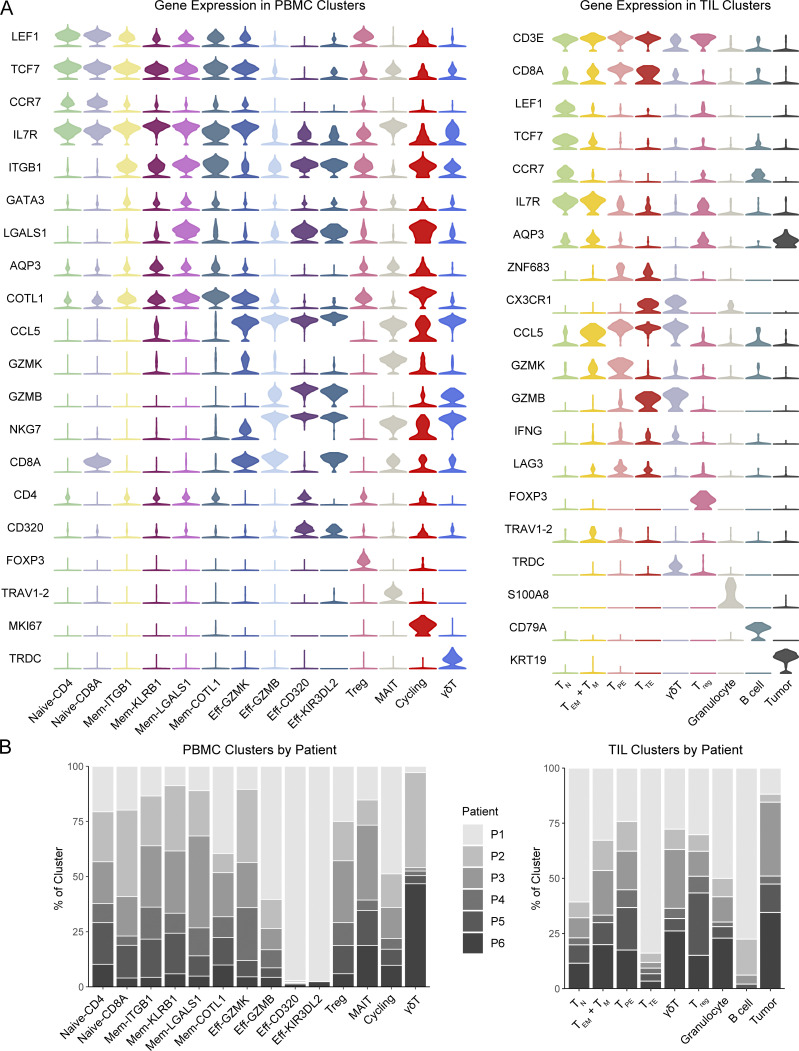
**scRNA-seq of patient PBMCs and TILs captures various circulating and tumor-infiltrating T cell subsets. (A)** Violin plots showing the log-normalized expression of cluster-defining genes across PBMC (left) and TIL (right) clusters. **(B)** Stacked bar plots showing the contribution of each patient’s cells to PBMC (right) and TIL (left) clusters. T_N_, naive T cells; T_M_, memory T cells; T_EM_, effector memory T cells; T_PE_, progenitor exhausted T cells; T_reg_, regulatory T cells; T_TE_, terminal exhausted T cells.

**Figure S2.**
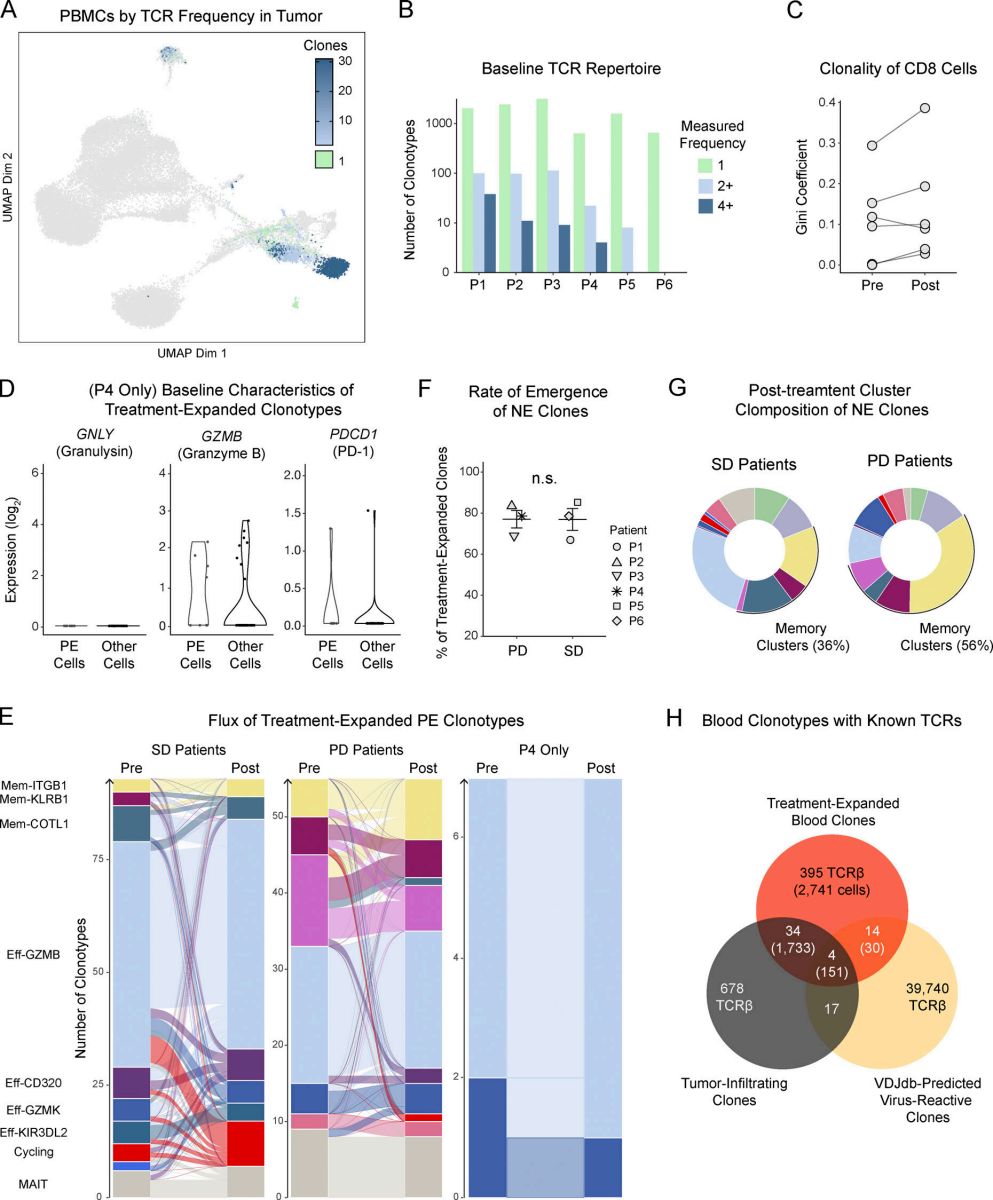
**Combined scTCR-seq and scRNA-seq of matched PBMCs and TILs enables characterization of treatment transcriptional effects on relevant clonal subsets. (A)** UMAP of circulating T cells where cells are colored by the observed frequency of their TCR in the matched tumor. **(B)** Bar plot showing the number of singleton, expanded, and hyper-expanded clonotypes by patient at the start of treatment. **(C)** Gini coefficient as a measure of clonality among CD8 cells before and after treatment with ribociclib and anti–PD-1. **(D)** For the single ovarian cancer patient P3, log-normalized expression of key genes in pre-treatment cells belonging to clonotypes that expanded after treatment in comparison to pre-treatment cells belonging to clonotypes that were expanded at baseline but did not expand further after treatment. Each dot represents a cell. Wilcoxon rank-sum test. **(E)** Alluvial plots showing clonotype flux from pre-treatment to post-treatment clusters; the thickness of a band connecting two clusters represents the number of clonotypes whose member cells were found in the corresponding clusters. SD refers to patients whose treatment response was stable disease for < 6 mo. PD denotes patients with progressive disease. P3 is the ovarian cancer patient. **(F)** Dot plot comparing the frequency of novel expanded clones in post-treatment samples between the two treatment response groups. Each dot represents a patient. Wilcoxon rank-sum test. **(G)** Donut plots showing the cluster identities of novel expanded clones stratified by treatment response. **(H)** Venn diagram showing clonotypes in our dataset predicted by VDJdb to be reactive to viruses using TCRβ-chain matching; numbers within parentheses represent the number of blood T cells expressing the indicated TCRβ chains. ****P < 0.0001.

In total, we sequenced 30,088 T cells and identified 22,433 distinct TCR clonotypes in the patients’ blood samples (Fig. 1, B and C). Cells were visualized by the similarity of their transcriptional profile on a Uniform Manifold Approximation and Projection (UMAP) plot and were found to cluster into 14 naive, effector, memory, and invariant subpopulations with cluster defining genes consistent with each of these cell types (Fig. S1 A). Cells from all patients were represented uniformly in most clusters (Fig. S1 B), with the exception of clusters Eff-CD320 and Eff-KIR3DL2, which were predominantly composed of patient-specific clonally expanded cells. Cluster Eff-CD320, in particular, consisted of a terminally differentiated effector CD4 population marked by high expression of cytotoxic genes and was almost entirely composed of an impressively expanded clonotype isolated from patient P1, putatively reactive to the tumor neoantigen NY-ESO-1 by sequence homology (Chen et al., 2021). Although the baseline T cell repertoire of the patients varied greatly in richness and clonal expansion (Fig. S2, A and B), most patients showed increased clonal expansion post-treatment (Fig. S2 C).

We first set out to determine whether we could detect treatment-induced effector cell expansion, the canonical effect of PD-1 blockade, which is known to be measurable not only in the tumor but also in peripheral circulation (Kamphorst et al., 2017; Zhang et al., 2020). Indeed, we found that many clonotypes from each of the six patients expanded in post-treatment blood samples relative to their pre-treatment frequency (Fig. 1 D), with the majority comprising CD8 T cells. Importantly, 39 of the 374 (10.4%) expanded clonotypes were also found in the tumor. The treatment-associated increase in clonal expansion disproportionately favored a subset of the sampled circulating repertoire. This pattern of increased clonal dominance, which occurred in five of the six patients, has previously been noted as an early signature of peripheral T cell dynamics in the setting of immune checkpoint blockade (Fig. S2 C; Valpione et al., 2020).

Differential expression analysis revealed that the source cells of treatment-expanded clonotypes expressed higher levels of *PDCD1* (coding for PD-1) and key cytotoxic genes (*GZMB*, *PRF1*, *GNLY*) relative to other pre-treatment cells, consistent with the preferential targeting of PD-1^+^ cytotoxic T cells by anti–PD-1 therapy (Fig. 1 E). We then examined the flux of treatment-expanded clonotypes from pre-treatment to post-treatment clusters, and we found that the majority of these clonotypes originated from the effector clusters Eff-GZMB, Eff-GZMK, and Eff-CD320 (Fig. 1 F), in keeping with differential expression analysis. The most frequent transcriptional states after treatment corresponded to the same set of effector clusters, with a notable transition of some clones into the Cycling-MKI67 cluster, indicative of ongoing clonal proliferation at the time of sampling. In subgroup analyses, we had insufficient PE cells from the patient with ovarian cancer to power any conclusions about differences driven by disease (Fig. S2, D and E), though, interestingly, the group of patients with briefly stable disease displayed more pronounced flux into the cycling cluster than patients with progressive disease (Fig. S2 E).

Interestingly, most treatment-expanded clonotypes were not detected in pre-treatment samples (Fig. 1 D). The emergence of novel expanded (NE) clonotypes, a phenomenon variously termed “clonal replacement” or “clonal revival,” has previously been associated with PD-1 blockade in several cancer settings (Liu et al., 2021; Yost et al., 2019). Conjecturing that some NE clonotypes are liable to be false-positive artifacts of the inevitable undersampling of the T cell repertoire, we compared our findings to a published dataset of longitudinal peripheral scTCR-seq in metastatic breast cancer patients receiving standard of care chemotherapy with or without the PD-L1 blocking antibody atezolizumab (Zhang et al., 2021). We found that the rate of emergence of NE clonotypes was significantly elevated over the background rate observed in chemotherapy-only controls (Fig. 1 G). TCR-seq depth was similar across the three compared treatment groups (Fig. 1 H).

**Figure 2.**
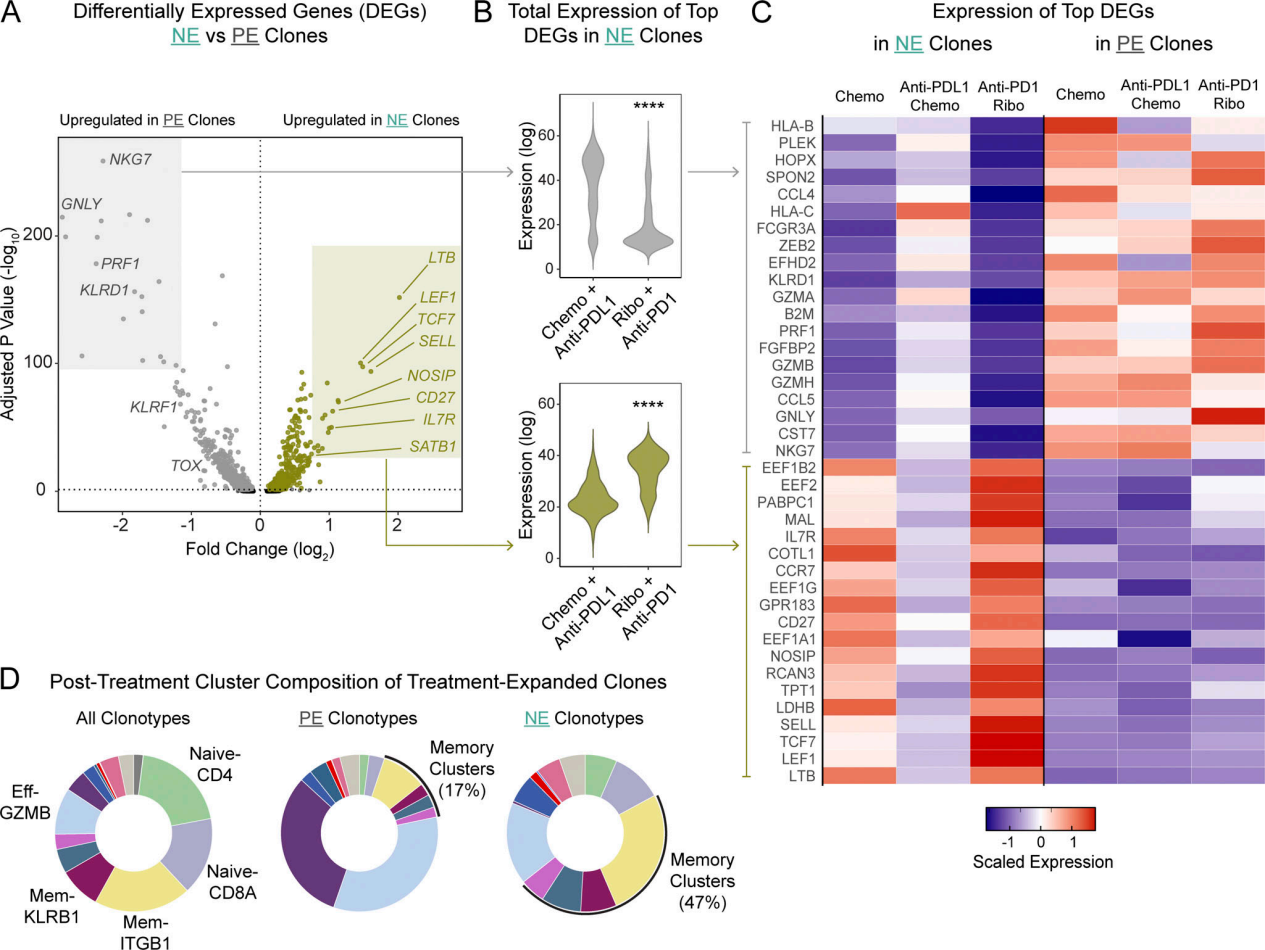
**Novel expanded clones arising upon blockade of the PD-1/PD-L1 axis exhibit enhanced memory and stem-like features only when treatment with ribociclib is added on.** PBMCs from metastatic breast or ovarian cancer patients investigationally treated with ribociclib and anti–PD-1 were sorted for CD3^+^ cells and analyzed by scRNA-seq and scTCR-seq (*n* = 6). Cells expressing the same TCR sequence were grouped into clonotypes and tracked over time. **(A)** Volcano plot showing differentially expressed genes between novel expanded clonotypes and pre-existing treatment-expanded clonotypes. Each dot represents a gene. Statistical testing was done using MAST. **(B)** Total normalized expression within novel expanded clonotypes of the top 20 marker genes and bottom 20 marker genes shown in A; expression levels are compared between patients who received PD-1/PD-L1 blockade with or without CKD4/6 inhibition. Wilcoxon rank-sum test. **(C)** Heatmap showing *z*-scaled log-normalized expression of the top 40 genes that distinguished NE clonotypes from PE clonotypes in ribociclib + anti–PD-1 patients; values are scaled by row across treatment groups. **(D)** Donut plots showing the cluster composition of post-treatment cells belonging to PE clonotypes (center) and NE clonotypes (right). ****P <0.0001.

We proceeded to examine the transcriptomes of NE clonotypes, reasoning that they would be enriched with recently naive T cells that had newly encountered their antigen during the study interval. Our group and others have previously demonstrated that exposing recently activated T cells to a CDK4/6 inhibitor, whether in vitro or in vivo, skews them toward a memory fate (Heckler et al., 2021; Lelliott et al., 2021). Thus, we hypothesized that we could detect evidence of this phenomenon within NE clonotypes. Differential expression analysis of NE clones versus pre-existing expanded (PE) clones showed strong upregulation of genes associated with memory and stem-like phenotypes (*TCF7*, *IL7R*, *CD27*, *LEF1*, and *SATB1*), whereas genes associated with exhaustion (*TOX*; Alfei et al., 2019; Khan et al., 2019; Scott et al., 2019) and terminal effector function (such as *NKG7* and *GNLY*) were relatively downregulated (Fig. 2 A). To determine whether this effect usually occurs with blockade of the PD-(L)1 axis or whether the memory skewing effect is a result of the ribociclib combination therapy, we extracted the top 40 differentially expressed genes between NE and PE clones and measured their expression in the Zhang et al. dataset (Zhang et al., 2021). We confirmed that the memory-skewed markers of NE were uniquely upregulated in patients treated with anti–PD-1 and ribociclib, while treatment with anti–PD-L1 strongly favored upregulation the effector-rich markers of PE cells (Fig. 2, B and C). This distinct transcriptional profile was reflected in the cluster distribution of NE clones, which were found more frequently in memory clusters than PE clones (Fig. 2 D), an effect that was preserved regardless of the patient’s clinical outcome (Fig. S2 G).

**Figure 3.**
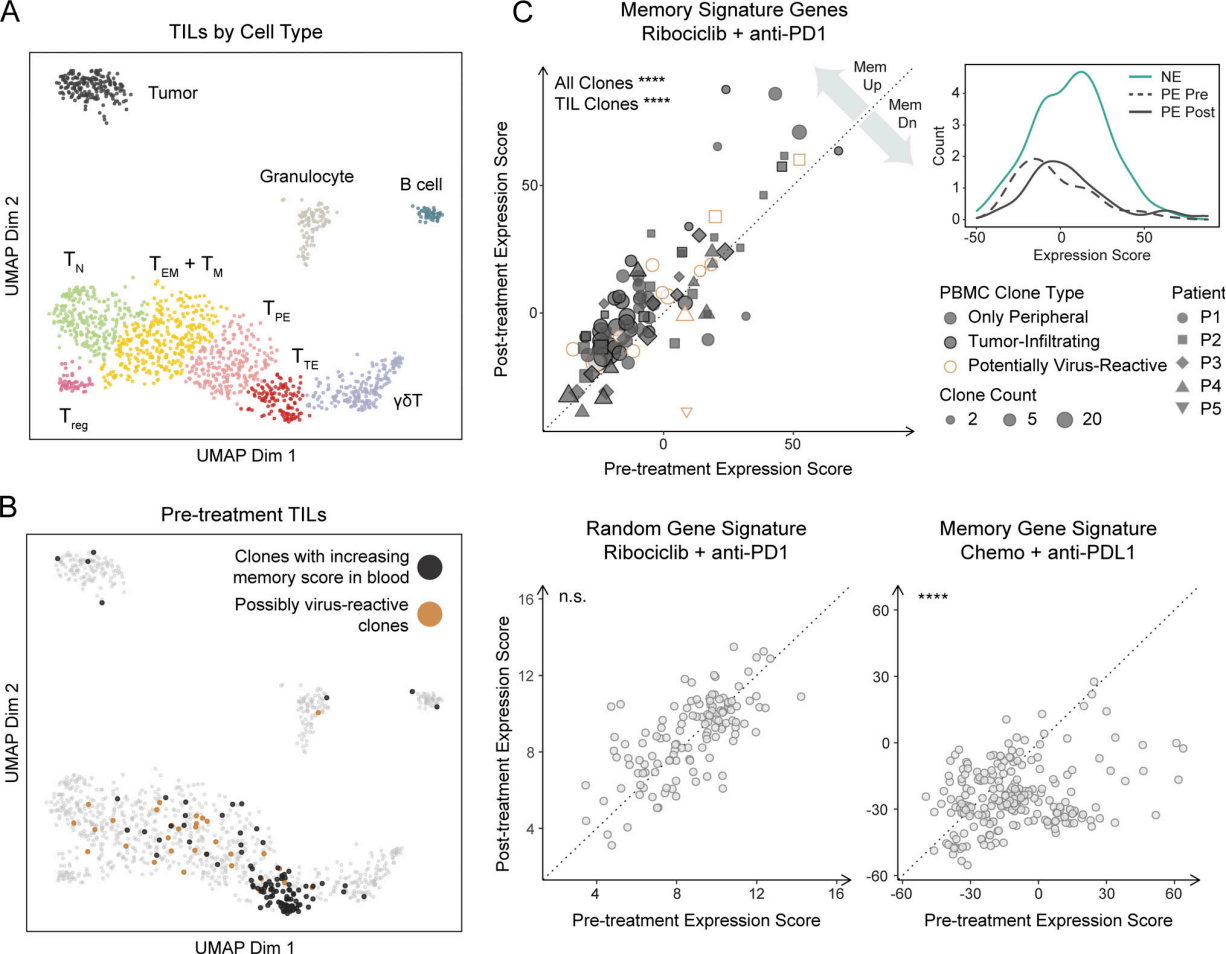
**Ribociclib-associated memory augmentation benefits tumor-infiltrating clonotypes.** Tumor-infiltrating cells from metastatic breast or ovarian cancer patients investigationally treated with ribociclib and anti–PD-1 were sorted for CD45^+^ cells and subject to scRNA-seq and scTCR-seq (*n* = 6). **(A)** UMAP of cells colored by cluster. T_N_, naive T cells; T_M_, memory T cells; T_EM_, effector memory T cells; T_PE_, progenitor exhausted T cells; T_reg_, regulatory T cells; T_TE_, terminal exhausted T cells. **(B)** UMAP of pre-treatment tumor cells highlighting clones that experienced an increase in memory gene expression in the circulation and clones that were predicted to be virus-reactive. **(C)** The expression of a set of genes upregulated in memory vs. effector precursor cells was measured in each circulating T cell clonotype before and after treatment; highlighted are circulating clones with matching counterparts detected in the tumor and circulating clones predicted to be virus-reactive (top left); the distributions of memory signature scores in novel expanded clonotypes and in pre-existing clonotypes before and after treatment (top right); the expression of a random set of genes was measured in our dataset to validate the absence of a batch effect between timepoints (bottom left); the expression of the memory gene set was measured in a control dataset of patients treated with PD-L1 blockade (bottom right). Wilcoxon signed-rank test. ****P < 0.0001.

We next analyzed baseline tumor biopsies and identified several transcriptionally defined clusters of T cells including naive, memory/effector memory, progenitor exhausted, and terminal exhausted, similar to previous reports (Lowery et al., 2022; Oliveira et al., 2021; Fig. 3 A). We cross-compared all TCR clonotypes from tumor and blood to a database of viral reactive clonotypes and found that 3.4% of clonotypes had putative viral specificities (Bagaev et al., 2020; Fig. S2 H). Consistent with previous findings, these possibly viral reactive clonotypes were predominantly found in the memory/effector memory T cell tumor-infiltrating clusters (Caushi et al., 2021; Oliveira et al., 2021; Fig. 3 B).

**Figure S3.**
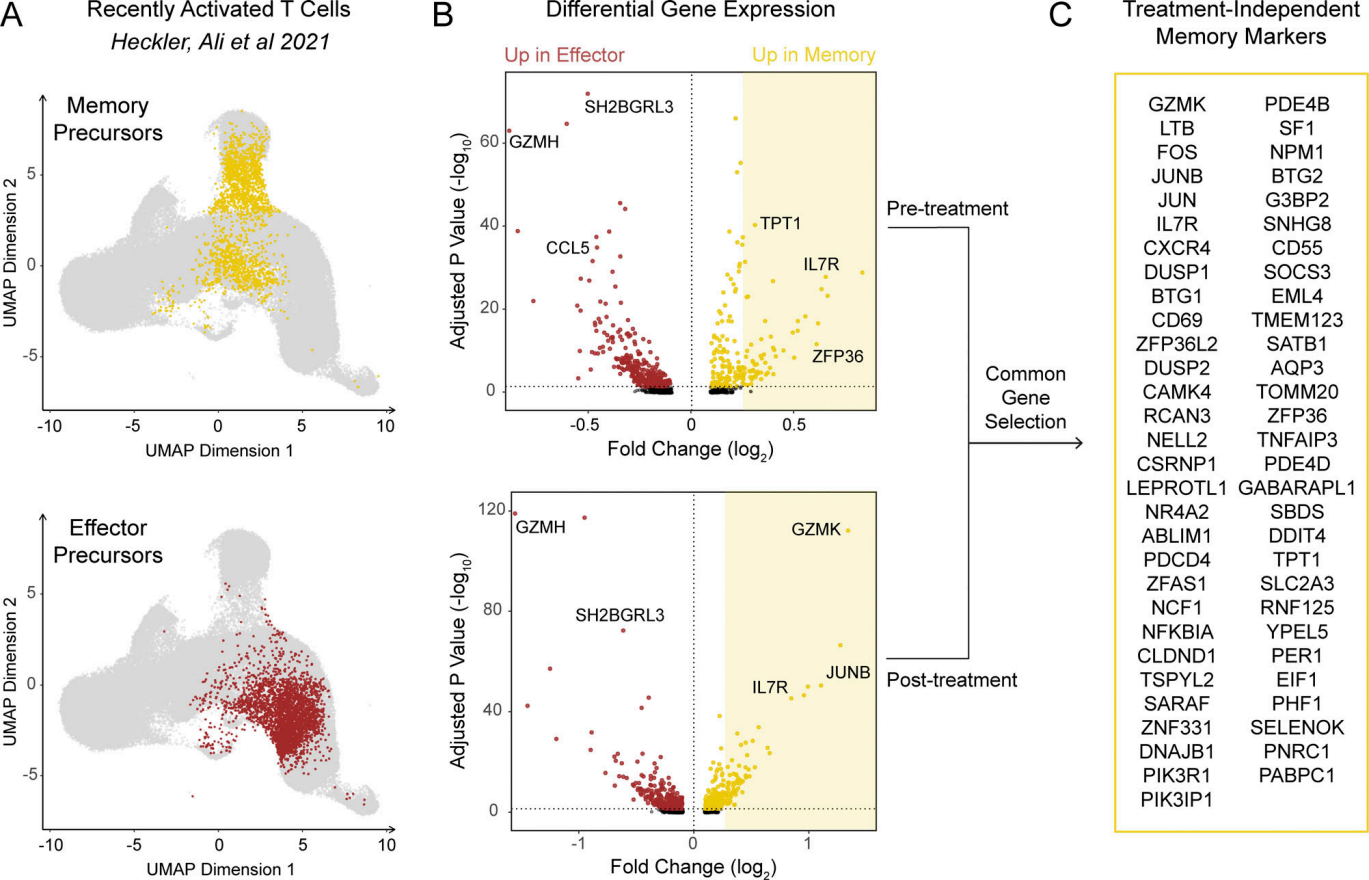
**Differential expression analysis between memory and effector precursor T cells generates a memory-associated marker gene set.** Recently activated (CD45RA^+^ CD45RO^+^) CD8 T cells from breast cancer patients before and after treatment with a CDK4/6 inhibitor were subject to scRNA-seq as described in a previously published dataset. **(A)** UMAP of cells highlighting memory (top) and effector (bottom) precursor cells. **(B)** Volcano plots of differentially expressed genes between memory and effector precursors in pre-treatment samples (top) and post-treatment samples (bottom); only genes from the shaded area (log-fold change >0.25) were considered for further analysis. **(C)** List of genes that appeared in differential expression at both timepoints and constituted the memory gene signature used in our analysis.

We next sought to investigate whether only NE clonotypes experienced an upregulation of memory markers or whether all non-singleton clonotypes exhibited some degree of memory skewing among their member cells. To this end, we took advantage of a transcriptional signature that differentiated memory from effector precursors among recently activated T cells in our previously published dataset (Fig. S3). Each clonotype was assigned a memory score before and after treatment. Most PE clonotypes (80%) scored modestly higher at the latter timepoint, consistent with these clonotypes mostly being terminally differentiated cells (Fig. 3 C). This effect was reversed in the Zhang et al. dataset, indicating that PD-L1 plus chemotherapy is associated with a decreased memory signature (Fig. 3 C). Scoring with an irrelevant gene signature showed no significant change between pre- and post-treatment samples (Fig. 3 C). Importantly, after cross-matching TCR sequences to TILs screening out putative viral-reactive clonotypes, clonotypes matching those in blood with increasing memory scores were predominantly found in the terminal exhausted T cell cluster, suggesting that at least some of the T cell clonotypes with increased memory skewing are tumor-specific (Fig. 3 B; Oliveira et al., 2021).

**Figure 4.**
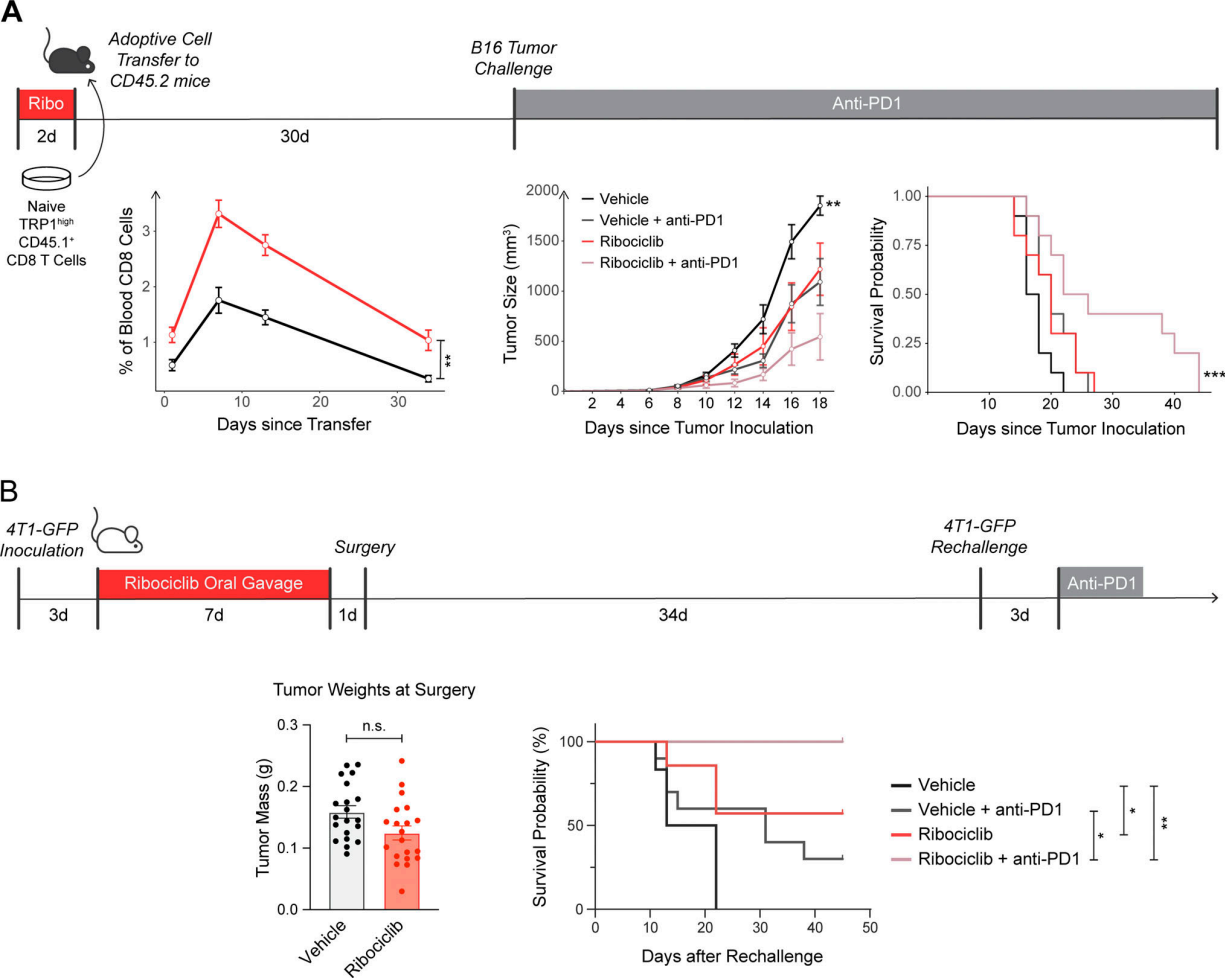
**Early treatment of tumor-reactive T cells creates a superior cell substrate for subsequent anti–PD-1 therapy in mice. (A)** CD8^+^ cells were collected from the spleen and lymph nodes of TRP1^high^ CD45.1^+^ transnuclear mice and activated in vitro with anti-CD3/CD28 beads in the presence or absence of ribociclib. They were then adoptively transferred into C57BL/6 mice (2 × 10^6^ cells per mouse, *n* = 20 per group). The frequency of CD45.1^+^ was tracked by flow cytometry on serial bleeds (left). The mice were rested then challenged with 3 × 10^5^ B16F10 tumor cells on day 37 by s.c. injection. Half of each mouse cohort was randomized to receive PD-1 blockade. Tumor size was tracked (middle) and survival was measured as time to reach size endpoint criteria or tumor ulceration (right). Blood CD8s and tumor sizes were compared to the vehicle group using a Wilcoxon rank-sum test. Survival was analyzed using a Cox proportional hazards regression model, with the comparison against the vehicle group shown. **(B)** Female BALB/c mice were inoculated with 200,000 4T1-GFP tumor cells near the mammary fat pat and treated with ribociclib (150 mg/kg) or vehicle by oral gavage from day 3 to 10 post-inoculation. Tumors were surgically removed at day 11 and primary tumor weights are shown. Mice were rested for an additional 34 d prior to rechallenge with 400,00 4T1-GFP tumor cells. Anti–PD-1 (150 µg/mouse) or isotype control was administered 3 d after rechallenge and mice were followed for survival. Tumor weights are representative of two independent experiments. Survival is combined from two independent experiments. Vehicle: *n* = 6; vehicle + anti–PD-1: *n* = 10; ribociclib + isotype: *n* = 7; ribociclib + anti–PD-1: *n* = 4. P values were determined by log-rank Mantel-Cox test. *P < 0.05, **P < 0.01, ***P <0.001.

Motivated by the finding that ribociclib may promote memory formation among tumor-infiltrating clones in patients, we wondered if early treatment with a CDK4/6 inhibitor could “sensitize” patients to later therapy with PD-1 blockade by providing a superior pool of antitumor memory cells. Although sequential therapy has been tested in mice and humans, none of the previous studies separated drug dosing by enough time to allow the formation of memory cells. To determine whether memory cells formed in the presence of CDK4/6 inhibition could be reinvigorated by PD-1 blockade, we used a murine model of melanoma. CD45.1^+^ TRP1^high^ CD8 T cells, whose TCR recognizes the melanoma antigen TRP1 with high affinity (Dougan et al., 2013), were adoptively transferred into CD45.2^*+*^ mice after being activated in vitro in the presence or absence of ribociclib. The mice were rested for 36 d, during which time the frequency of CD45.1^+^ T cells in their blood was measured weekly. The cells that had been activated in the presence of ribociclib expanded and persisted to a greater degree (Fig. 4 A). On day 37, the mice were challenged with tumors, and half of each group was randomly selected to receive anti–PD-1 therapy. Only those mice that had received ribociclib-treated cells experienced a significant survival benefit from PD-1 blockade (median survival of 24 vs. 17 d, P = 0.0003, Fig. 4 A).

To test whether oral dosing of ribociclib to a whole mouse could similarly affect endogenously generated anti-tumor T cells, we used a 4T1 murine breast cancer cell line that had been transduced to express GFP. On the BALB/c background, GFP is presented by H-2K^d^ and can induce a robust CD8 T cell response (Agudo et al., 2015; Krall et al., 2018). We implanted BALB/c mice with 4T1-GFP tumor cells and treated the mice for 1 wk with daily ribociclib by oral gavage. This short course of ribociclib reduced tumor size but did not clear the tumors. All mice were then surgically cured and allowed to rest for 35 d to recover from surgery and allow for memory cell development. Mice were then rechallenged with twice the dose of 4T1-GFP tumor cells on the opposite flank and randomized to receive either anti–PD-1 or isotype control. As shown in Fig. 4 B, mice receiving either ribociclib or PD-1 blockade alone showed increased survival compared to mice receiving no active therapy. However, mice that had received an early course of ribociclib and later received PD-1 blockade were fully protected, with significantly increased survival compared to PD-1 blockade alone. These data show that, in a mouse model of breast cancer, neoadjuvant ribociclib alone induced immunologic memory, and that outcomes were further enhanced with sequential checkpoint blockade.

Prior to this study, attempts at dual immunotherapy with a CDK4/6 inhibitor and PD-1 blockade in metastatic cancer did not show promise, largely due to frequently treatment-limiting toxicities (Pujol et al., 2021; Rugo et al., 2020). By retrospectively analyzing circulating and tumor-infiltrating T cells from treated patients, taking advantage of single-cell-resolution sequencing to track clonal dynamics and transcriptomes over time, we were able to gain deep insights into the cooperative but nonoverlapping effects of each treatment on reshaping patients’ antitumor immune response. We verified that cell-cycle inhibition by ribociclib does not prevent the desirable effect of PD-1 blockade on cytotoxic effector cell expansion. We also replicated the finding of memory fate skewing under the influence of CDK4/6 inhibition in this setting, and extended the significance of this observation by matching the affected circulating cells to exhausted T cell populations in the tumor.

PD-(L)1 blockade has been shown to reinvigorate a stem-like progenitor population of exhausted CD8 T cells in both mice and humans (Beltra et al., 2020; Collier et al., 2021; Eberhardt et al., 2021; Hudson et al., 2019). The origin of these stem-like progenitor cells likely involves newly primed T cells as new T cell priming and replacement of clonotypes is simultaneously occurring with PD-(L)1 blockade (Liu et al., 2021; Yost et al., 2019). Single-cell analysis from breast cancer patients has shown that intratumoral CD103^+^ resident memory T cells correspond with improved prognosis, although treatment with PD-1 blockade appears to reinvigorate a different population of CD8 effector memory cells characterized by expression of *GZMK* and *EOMES* (Bassez et al., 2021; Savas et al., 2018). Whereas PD-(L)1 blockade pushes T cells toward a more differentiated effector cell fate, we have shown that the addition of ribociclib skews newly primed CD8 T cells toward a memory-like fate, thereby replenishing the stem-like progenitor cell pool.

A major strength of our methodology was the deployment of detailed scTCR clonotype tracking, an innovative expansion of scRNA-seq technology. Global increases in the human T cell memory pool do not occur with CDK4/6 inhibitors (Peuker et al., 2022), precluding use of bulk assays. Our work highlights the power of this platform, which enabled us to isolate specific subgroups of clonally expanded populations (PE vs. NE clones) that were predicted to be differentially affected by combination treatment. Parsing out distinct transcriptional signatures of treatment would have been impossible with traditional single-cell analyses in which cells are clustered and compared on the basis of their transcriptional similarity.

Our analysis was limited by low recovery of TILs, which likely caused us to miss some tumor-matched circulating clonotypes. While we speculate that memory skewed T cells in the blood would migrate into the tumor, we were limited by lack of adequate paired on-treatment biopsies to determine the fate of treatment-expanded clonotypes in the tumor itself. Additionally, some clonotypes that we label as potentially tumor-reactive may not be, given that definitive epitope determination is not possible by TCR sequencing alone. Furthermore, it is important to note that we limited our analytical focus to pre-existing knowledge of the signature effects of each treatment. Our dataset originated in an early, safety-oriented clinical trial which did not compare combination therapy to single-agent therapy controls. As such, we are unable to assess novel or unexpected effects that may arise in the combination treatment setting. Although our cohort included one patient with ovarian cancer who did not receive fluvestrant, we recognize that this is insufficient to exclude potential confounding effects of cancer type or administration of selective estrogen receptor degraders.

Despite these limitations, we believe that our observations motivate further study of combined but sequential therapy with CDK4/6 inhibitors and PD-1 blockade. While the introduction of immune checkpoint blockade has been a revolutionary step in the treatment of many cancers, only a minority of patients benefit. Our findings suggest that early exposure to CDK4/6 inhibitors may generate a critical pool of memory T cells to act as the foundation of subsequent anti–PD-1 responses. Since none of our patients achieved clinical benefit, we speculate that these differences in T cell biology may be even more pronounced in patients responding to therapy. Recent work supports the plausibility of this paradigm, as persistent neoantigen-reactive T cells that arise after administration of tumor vaccines appear to robustly reinvigorate with PD-1 blockade (Hu et al., 2021). Simultaneous combination therapy of CDK4/6 inhibitors with PD-1 or PD-L1 blockade have unacceptably high rates of hepatotoxicity; however staggering use of these regimens in sequential fashion offers a chance to avoid toxicities while still retaining much of the immune-potentiating effects of CDK4/6 inhibitors. Therefore, early sequential application of CDK4/6 inhibitors, perhaps in the neoadjuvant setting, followed by checkpoint inhibition could become a key strategy in increasing the fraction of the population responsive to PD-1 blockade.

## Materials and methods

### Patient sample preparation

Peripheral blood monocytic cells (PBMCs) were isolated from the blood of breast or ovarian cancer patients enrolled in a clinical trial at the Dana-Farber Cancer Institute (DFCI; NCT03294694) using Ficoll gradient centrifugation. TILs from biopsies of the primary site or metastatic sites were also obtained. Consent for collection of the samples and general use in research was included in the clinical trial protocol, which received Institutional Review Board approval by the Dana-Farber/Harvard Cancer Center Office for Human Research Studies under protocol number 17-285; specific Institutional Review Board approval for our secondary-use analysis of patient samples was provided under protocol number 21-590. All cells were stained with the Zombie NIR live/dead stain (#423105; BioLegend) and the following fluorophore-conjugated antibodies: anti-CD45 PacificBlue (clone HI30; #304029; BioLegend), anti-CD3 PE-CF594 (clone UCHT1; BD #562280), anti-CD14 APC (clone HCD14; #325608; BioLegend), anti-CD16 APC (clone 3G8; #302012; BioLegend), and anti-CD19 APC (clone 1D3/CD19; #152409; BioLegend). In addition, a “hashtag” antibody bound to a unique RNA barcode was added to each sample separately during staining to allow for downstream multiplexing (BioLegend TotalSeq-C0251, -C0252, -C0253, and -C0254). Samples were then sorted on a BD FACS Aria II SORP machine, selecting for live CD45^+^ CD3^+^ CD14^−^ CD15^−^ CD19^−^ cells from blood and live CD45^+^ cells from tumor.

### scRNA-seq

Sorted single-cell suspensions prepared from each sample were washed twice with 0.05% UltraPure BSA (#AM2618; Invitrogen) in PBS. For PBMC samples, 6,000 cells were loaded into a 10× Chromium controller instrument along with Chromium Next GEM Single Cell 5′ beads (10× Genomics PN-1000263). Up to four PBMC samples were multiplexed together after being tagged with unique RNA tags as described above. For TIL samples, all sorted cells (500-2,000) were loaded, and no multiplexing was performed. After RT-PCR, cDNA was purified, and a library was constructed from each sample using a 10× Library Construction Kit (10× Genomics PN-1000190) following the standard 10× protocol. An additional VDJ-enriched library was created for each sample using a specialized Chromium Single Cell Human TCR Amplification Kit (PN-1000252). Libraries were then sequenced on an Illumina HiSeq system operated by Azenta/Genewiz generating paired-end 150 bp reads.

### Data analysis

The 10× CellRanger “multi” pipeline (v6.0.1) was used to align reads to the GRCh38 reference genome and generate a single-cell feature count matrix for each library using default parameters. The count matrices were imported for downstream analysis into R using the “Seurat” package (v4.0.03). Genes with zero expression across all cells were discarded from further analysis. PBMC cells with >20% mitochondrial reads were discarded. For tumor samples, a 30% cutoff was used. Data from each sample were log-normalized and combined into one batch-corrected expression matrix by Canonical Correlation Analysis. Counts were then scaled and subject to dimensionality reduction using Principal Component Analysis. UMAP embedding was generated from the top 25 dimensions of the Principal Component Analysis for PBMC samples and 45 dimensions for tumor samples. Clusters were identified first by constructing a Shared Nearest Neighbor graph based on each cell’s *k*-nearest neighbors (*k* = 20 for PBMCs, *k* = 15 for TILs) and then applying the Smart Local Moving algorithm to the graph. Markers for each cluster were identified by comparing gene expression using Model-based Analysis of Single-cell Transcriptomics (MAST).

### Clonotype identification and tracking

T cell clonotypes were identified by the 10× CellRanger software for PBMC and TIL samples separately. Matching of clonotypes between blood and tumor was performed by matching the amino acid sequence of both the α-chain complementarity determining region 3 (CDR3) and the β-chain CDR3. A minority of clonotypes lacked information for either the α or the β TCR chain, and for those, matching on only one chain was permitted.

### Comparative analysis with Zhang et al. (2021)

The scRNA count matrix was obtained from GEO under the accession GSE169246. We cross-correlated the counts with scTCR data provided as supplemental information with the original publication. We only considered the subset of the count matrix that corresponded to those cells with identified TCR sequences in the blood. The resulting matrix was merged with our own dataset’s RNA count matrix and log-normalization was than done in Seurat as described above.

### Memory signature score

Differential expression analysis was performed on a peripheral T cell scRNA-seq dataset previously published by our group (dbGaP accession phs002448.v1.p1). Memory precursor cells (belonging to the cluster originally labeled as 5M) were compared using MAST to effector precursor cells (cluster 5E) separately within pre-treatment samples and within post-treatment samples. Genes that were upregulated in memory cells at both times were selected as a treatment-independent memory gene signature. Genes with a log fold change (LFC) below 0.25 at both timepoints were discarded, as were any mitochondrial or ribosomal genes. In addition, a weight was assigned to each gene using the antilog of the average of its pre-treatment LFC and post-treatment LFC. Each cell was assigned a *z* score for each signature gene based on the global log mean expression of that gene. Finally, the memory signature score of a given clonotype was calculated as the weighted sum of the average *z* score of all signature genes among the cells belonging to that clonotype.

### Animal care

Animals were housed at the DFCI and were maintained according to protocols approved by the DFCI Institutional Animal Care and Use Committee (#14-019 and #14-037). TRP1^high^ transnuclear mouse lines were generated by us as previously reported and maintained in house (Dougan et al., 2013). This line is now available through Jackson Labs (stock #30958). TRP1^high^ mice were also crossed to CD45.1+ mice from Jackson Labs (B6.SJL-Ptprca Pepcb/BoyJ, stock #002014). C57BL/6 (stock #000664) and BALB/c (stock #000651) mice were purchased from Jackson Labs. Female mice were used throughout for both T cell donors and recipients to avoid immunogenicity of Y-chromosome encoded genes.

### Cell lines

B16-F10 cells were purchased from ATCC. 4T1-GFP cells were a gift from Dr. Judith Agudo (DFCI, Boston, MA, USA). Cells were cultured in RPMI 1640 medium (Gibco) supplemented with 10% heat-inactivated FBS (# FB-11; Omega Scientific catalogue), 2 mM L-glutamine (Gibco), penicillin G sodium (100 U/ml, Gibco), streptomycin sulfate (100 μg/ml; Gibco), 1 mM sodium pyruvate (Gibco), 0.1 mM nonessential amino acids (Gibco), and 0.1 mM β-mercaptoethanol (Sigma-Aldrich). Cells were passaged two to three times prior to use and were used for experiments at 80–90% confluency. Mycoplasma testing was performed by PCR every 4 mo and was negative for the entire course of this study. No further authentication was performed.

### Adoptive transfer

Spleens and lymph nodes were harvested from TRP1^high^ CD45.1^+^ mice. They were crushed through 40-μm cell strainers in PBS. CD8^+^ T cells were isolated by negative selection using an EasySep Mouse CD8^+^ T cell Isolation Kit (StemCell #19853). The cells were cultured in RPMI complete containing 100 U/ml human IL-2 (Peprotech 200-02-250UG) and CD3/CD28 Dynabeads (#11456; Gibco) with or without ribociclib at 200 nM. After 48 h, 2 × 10^6^ CD45.1^+^ CD8^+^ T cells in 150 μl of sterile PBS were transferred by tail vein injection into sex-matched CD45.2^+^ C57BL/6 recipient mice, with half of the mice receiving cells that had been activated in the presence of ribociclib. For the longitudinal monitoring of cell persistence after transfer, mice were bled in the indicated intervals, and flow cytometry was performed.

### Mouse blood flow cytometry

Blood samples from mice were depleted of red blood cells by washing with ACK lysis buffer. They were then stained with the following fluorophore-conjugated antibodies: anti-CD8 APC (clone 53-6.7, #100712; BioLegend) and anti-CD45.1 FITC (clone A20, #110706; BioLegend). For the day 18 timepoint after tumor challenge, a larger antibody panel was used: anti-CD8 PacificBlue (clone 53-6.7, #100725; BioLegend), anti-CD45.1 FITC (clone A20, #110706; BioLegend), anti-IL7Rα APC (clone A7R34, #135012; BioLegend), and anti-CX3CR1 PE (clone SA011F11, #149006; BioLegend). Antibodies were diluted in a staining buffer consisting of 2% heat-inactivated FBS in PBS, and staining was done at 4°C for 20 min. Samples were then analyzed on a Sony SP6800 Spectral Analyzer. Gating and analysis of flow cytometry data was done in FlowJo 10.8.1. Samples that yielded fewer than 200 CD8 cells were discarded from analyses requiring the estimation of the circulating frequency of CD8^+^ CD45.1^+^ cells.

### Tumor challenge

After 36 d of rest, C57BL/6 mice that had received adoptively transferred TRP1^high^ CD45.1^+^ CD8^+^ cells were inoculated with 3 × 10^5^ B16 tumor cells in sterile Hank’s Balanced Salt Solution by subcutaneous injection into the left flank. Mice were grouped based on whether they had received cells activated in the presence of absence of ribociclib, then a random half of each group was selected to receive a monoclonal antibody against PD-1 (clone RMP1-14, Bio X Cell BE0146) at 150 µg/mouse starting on day 4, when tumors became palpable. Dosing was done twice a week by intraperitoneal injection. Tumors were measured every other day along three dimensions by the same investigator, and volumes were estimated by the ellipsoid volume formula. Mice were sacrificed if the tumor reached 2,000 mm^3^ in volume, 20 mm in any dimension, or developed ulceration.

### 4T1-GFP neoadjuvant model

BALB/c mice were inoculated into a shaved area of skin near the lower mammary fat pad with 200,000 4T1-GFP tumor cells suspended in 100 μl of Matrigel mixed 1:1 with PBS. Tumors were allowed to grow for 11 d. From days 3–10, mice were treated daily by oral gavage with 150 mg/kg ribociclib, dissolved in 0.5% methylcellulose (Sigma-Aldrich) or with vehicle alone. At day 11, subcutaneous tumors measured no more than 8 mm in diameter. Mice were anesthetized with ketamine/xylazine. Meloxicam was administered pre-operatively. Shaved area was cleaned with iodine solution (betadine swabs) and ethanol. An incision (5–10 mm) was made in the skin directly above the tumor and the tumor was manipulated gently with forceps to release it from the skin and surrounding tissue. The tumor and a 1 mm margin of fat was excised with scissors. Minor bleeding was dabbed with sterile cotton swabs or cauterized if necessary. The incision was flushed with a 1:1 mix of lidocaine (did not exceed 10 mg/kg) and bupivacaine (did not exceed 6 mg/kg) for local pain relief. Two to three metal wounds clips were used to close the skin, depending on the size of the incision. Mice were placed on a heating pat and monitored until they recover from anesthesia. Meloxicam was administered every 24 h for 48 h, 1 dose post-surgery. Any mice that developed post-surgical complications or regrowth of the primary tumor within 1 wk suggesting inadequate surgical removal were excluded. After 35 d post-surgery, mice were rechallenged subcutaneously on the opposite flank with 400,000 4T1-GFP cells suspended in Matrigel. 3 d post-rechallenge, mice were administered anti–PD-1 (clone RMP1-14, BioXcell) or isotype (clone LTF-2, BioXcell) at 150 µg per mouse intraperitoneally. Mice were monitored three times per week for tumor size and euthanized when tumor size reached <1,000 mm^3^ or developed ulceration.

### Statistics

For differential expression analysis, testing was performed using MAST as described above, and P values were adjusted for multiple hypothesis testing using the Bonferroni method. Comparison of pre-treatment to post-treatment gene expression scores was done with a nonparametric Wilcoxon signed rank test. Survival analysis made use of a Cox proportional hazards regression model with treatment as the sole predictor. All other reported P values are the results of nonparametric Mann-Whitney tests. Statistical analysis was done in R (4.0.2).

### Online supplementary material

Fig. S1 shows key cluster-defining genes used to support the identification and labeling of PBMC and TIL clusters as well as the breakdown of each cluster’s cells by patient origin. Fig. S2 shows additional characterization of the TCR repertoires in the dataset and breaks down important findings by treatment response and cancer type. Fig. S3 describes the method by which the memory precursor gene signature was generated from our previously published dataset of recently activated circulating T cells in breast cancer patients.

## Data Availability

Single-cell sequencing data have been deposited in the National Institutes of Health (NIH) database GEO under accession GSE205589.
