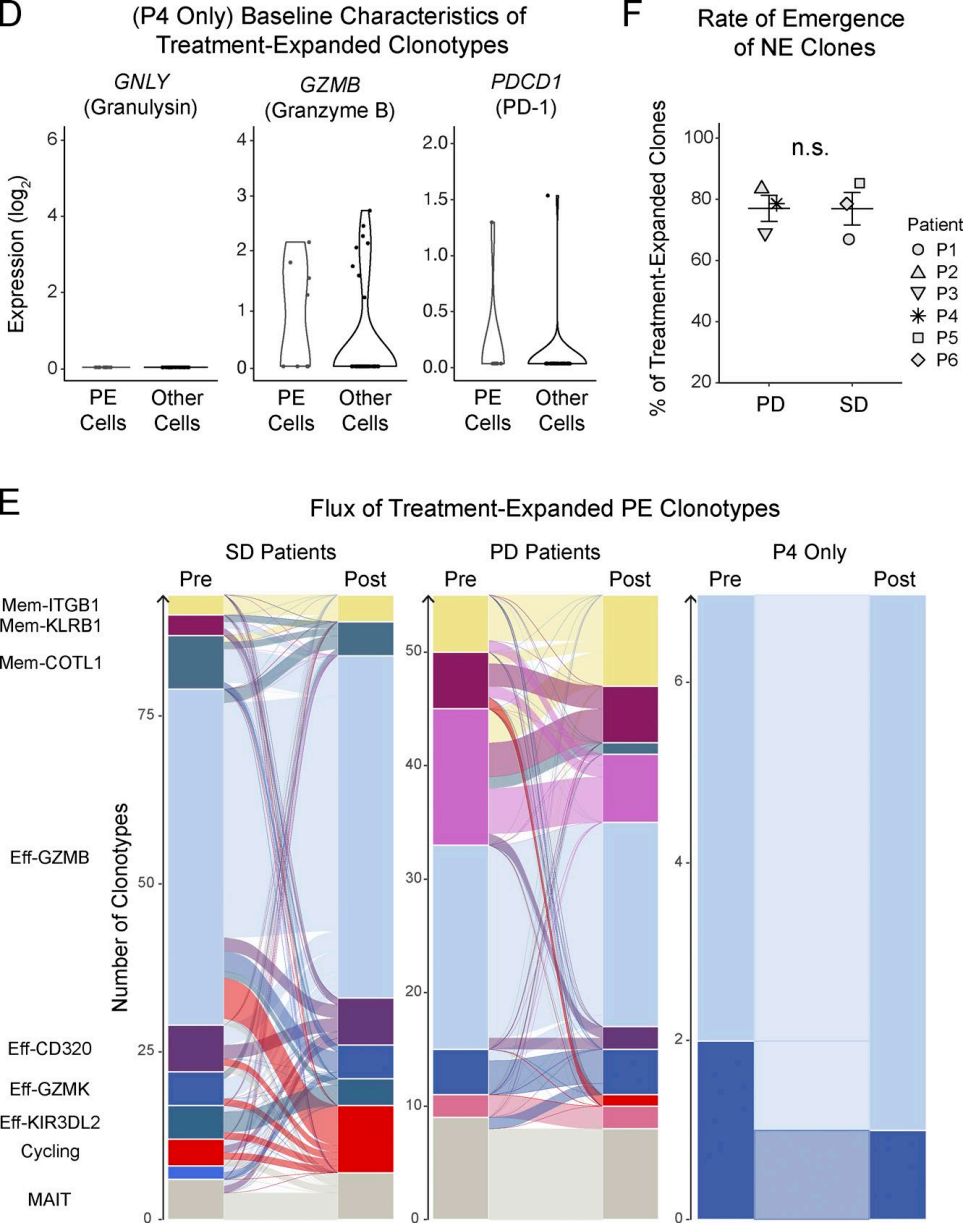# Correction: PD-1 blockade and CDK4/6 inhibition augment nonoverlapping features of T cell activation in cancer

**DOI:** 10.1084/jem.2022072908182023c

**Published:** 2023-08-24

**Authors:** Lestat R. Ali, Ana C. Garrido-Castro, Patrick J. Lenehan, Naima Bollenrucher, Courtney T. Stump, Michael Dougan, Shom Goel, Geoffrey I. Shapiro, Sara M. Tolaney, Stephanie K. Dougan

Vol. 220, No. 4 | https://doi.org/10.1084/jem.20220729 | January 23, 2023

This study included five patients with breast cancer and one patient with ovarian cancer. The authors regret that in the originally published article, the patient with ovarian cancer was incorrectly identified as “P3” due to an error while renumbering the internal patient identifiers. The patient with ovarian cancer is patient P4. This has been corrected in Fig. 1, D and G, in which the marker § now denotes P4. Fig. S2, D and E, has been replaced with data from patient P4, and the patient identifier numbers in F were updated for consistency. In addition, this patient had only eight pre-existing (PE) clonotypes, so the text at the end of the fourth Results and discussion paragraph has been modified as follows: “In subgroup analyses, we had insufficient PE cells from the patient with ovarian cancer to power any conclusions about differences driven by disease (Fig. S2, D and E).” This correction does not change the original conclusions of the manuscript, and the figure legends remain unchanged. The errors appear in print and in PDFs downloaded before August 18, 2023.

**Figure 1:**
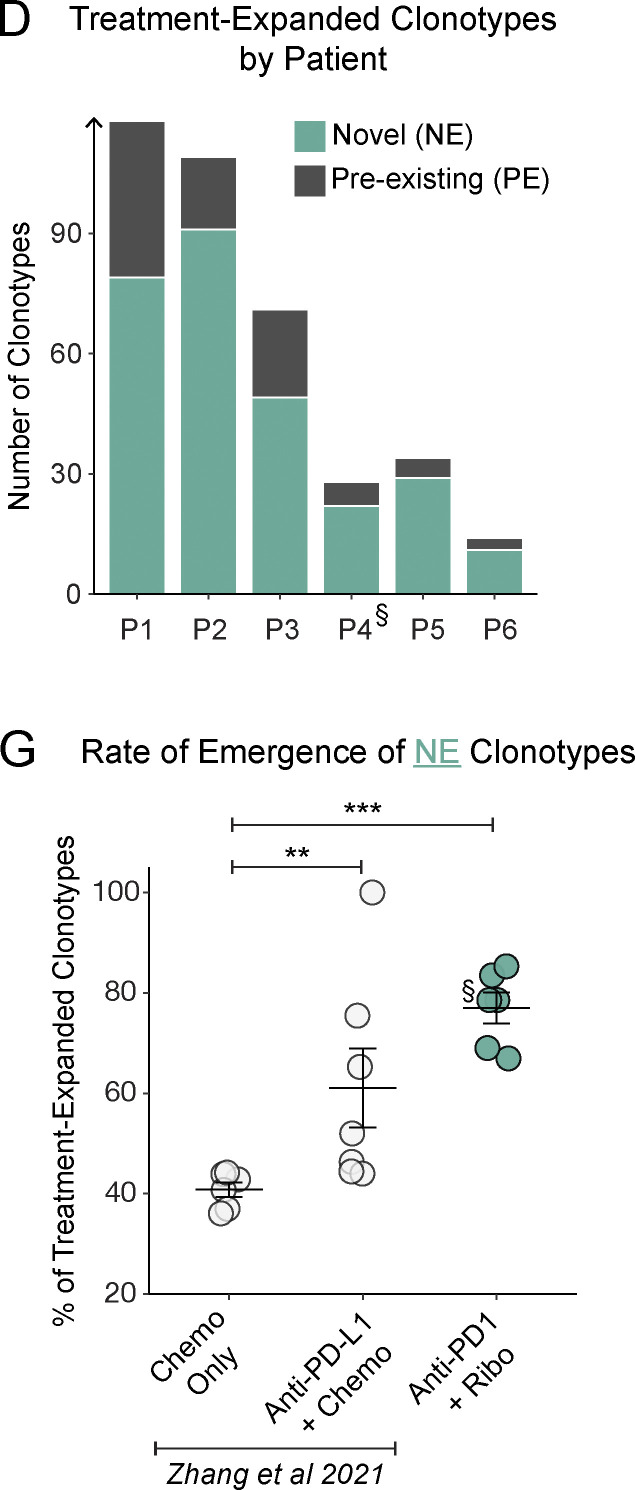


**Figure S2:**